# Hypo-connectivity of the primary somatosensory cortex in Parkinson’s disease: a resting-state functional MRI study

**DOI:** 10.3389/fneur.2024.1361063

**Published:** 2024-04-30

**Authors:** Yinghua Wang, Tao Gong, Na Tao, Ying Zeng, Haotian Ma, Wu Yuan, Wenmin Luo, Fuqing Zhou

**Affiliations:** ^1^Department of Radiology, The People's Hospital of Xiangzhou District, Zhuhai City, China; ^2^Department of Radiology, The People's Hospital of Yichun City, Yichun, China; ^3^Department of Neurology, The People's Hospital of Yichun City, Yichun, China; ^4^Department of Radiology, The First Affiliated Hospital, Jiangxi Medical College, Nanchang University, Nanchang, China

**Keywords:** the primary somatosensory cortex, connectomics, Parkinson’s disease, resting-state functional MRI, functional connectivity

## Abstract

**Background:**

Parkinson’s disease (PD) is characterized by a range of motor symptoms as well as documented sensory dysfunction. This sensory dysfunction can present itself either as a “pure” sensory disturbance or as a consequence of sensory-motor integration within the central nervous system. This study aims to investigate changes in the functional connectivity of the primary somatosensory cortex (S1) and its clinical significance in Parkinson’s disease (PD), an area that has received limited attention in previous neuroimaging studies.

**Methods:**

This study included thirty-three patients with PD and thirty-four healthy controls (HCs). Clinical evaluations were conducted to assess the clinical manifestations, severity, and functional capacity of all the patients. Resting-state functional MRI (fMRI) was employed to evaluate the functional connectivity of six paired S1 subregions in the participants. Seed-based correlation (SBC) analysis was utilized to construct the correlation matrix among the subregions and to generate connectivity maps between the subregions and the remaining brain voxels. Finally, the study employed partial least-squares (PLS) correlation analysis to investigate the association between modified functional connectivity and clinical characteristics in PD patients.

**Results:**

In the correlation matrix, patients with PD demonstrated a notable decrease in functional connectivity across various S1 subregions in comparison to HCs (*p* < 0.001, corrected using network-based methods). In connectivity maps, hypo-connectivity was primarily observed in the sensorimotor network as common patterns (*p* < 0.001, corrected for false discovery rate) and in the default mode network (DMN) as distinct patterns. Moreover, this study identified a negative association between the correlation matrix within S1 subregions and the scores for axial symptoms and postural instability/gait difficulty (PIGD) in PD patients. Nevertheless, a direct relationship between the connectivity maps of S1 subregions and clinical assessment scales was not established.

**Conclusion:**

This study offers novel insights into the neurobiological mechanisms that contribute to S1 dysfunction in PD, highlighting the significant involvement of S1 hypo-connectivity in the motor disturbances observed in PD patients.

## 1 Introduction

Parkinson’s disease (1) is a neurodegenerative disorder characterized by the degeneration of dopaminergic neurons in the substantia nigra, the formation of intracellular inclusion bodies known as Lewy bodies, and the accumulation of iron. Individuals diagnosed with PD typically present with four primary symptoms: rigidity, postural instability, bradykinesia, and tremor (2). Conventional magnetic resonance imaging is utilized as an adjuvant tool to rule out the existence of subcortical vascular pathology or other factors contributing to secondary parkinsonism (3). But, the conventional MRI lacks specificity and is inadequate for interpreting the symptoms of PD patients and making a diagnosis.

In recent years, a range of advanced neuroimaging techniques has been developed to offer valuable insights into the mechanisms that underlie PD (3–5). For instance, functional MRI (fMRI), a widely utilized technique in contemporary research, can be employed to identify variations in blood flow fluctuations and blood oxygen-level dependent signals in the brain. It can also be used to assess neuronal activation and/or intrinsic activity patterns by leveraging the paramagnetic properties of blood. In patients with PD, fMRI investigations have identified and validated disrupted functional connectivity (6) in the striatal, limbic, and parietal regions (7, 8), as well as significant effects on the sensorimotor network (SMN) (5, 9), basal ganglia network (BGN) (5), executive control network (ECN) (7), frontal-striatal network (10, 11), and default mode network (DMN) (12). Alterations in the motor-related brain network can elucidate the motor symptoms associated with dopamine in PD patients. Additionally, other changes in brain networks are also crucial in comprehending non-motor mechanisms, such as olfactory dysfunction (13, 14).

Multiple sensory impairments, such as proprioceptive, tactile, olfactory, visual, and auditory deficits, have garnered significant attention in PD patients (15, 16). Sensory dysfunction observed in PD can manifest as either “pure” disorders of perception or as disorders of sensorimotor integration, which are characterized by a breakdown in the sensory-regulated control of motor responses (15, 17, 18). Sensory abnormalities that affect motor function may consequently exacerbate the overall disease burden indirectly.

Recent advancements indicate that somatosensory deficits in PD patients may be attributed to dopaminergic denervation associated with the disease, leading to the transmission of more distorted and less distinct information to cortical regions (19). Spontaneous pain (20), sensory disturbances (19), and the modulation by deep brain stimulation (DBS) (21) have been associated with changes of PD in resting (or intrinsic) functional connectivity. Prior research has not comprehensively examined the primary somatosensory cortex (S1), where atypical spatial and temporal processing of sensory information could lead to the generation of inaccurate signals for the planning and implementation of voluntary movement. Hence, clarifying the neural bases of somatosensory abnormalities in PD could identify potential targets for neurointervention to enhance sensorimotor disturbances in PD. In this study, we hypothesized that abnormal S1 functional connectivity exists in PD patients and is associated with motor symptoms. The purpose of our study is to uncover the imaging neurobiological mechanisms of S1 in PD patients, and to study its relevance to PD-related disability, thereby revealing novel therapeutic possibilities.

## 2 Materials and methods

### 2.1 Participants

A total of 75 participants were recruited from the Department of Neurology at People’s Hospital of Yichun City between April 2020 and June 2022. The inclusion criteria for PD patients were as follows: (1) diagnosed according to the Movement Disorders Society (MDS) Clinical Diagnostic Criteria (22) and the Chinese Diagnostic Criteria for Parkinson’s Disease (2016 edition) (23), (2) right-handed and aged 48–81 years, and (3) *de novo* PD patients without medication. The HCs were recruited from the local community. The exclusion criteria for all participants were as follows: (1) the presence of confounding neurological diseases, vascular damage or cerebral infarction, (2) a history of impaired consciousness, manic episodes, schizophrenia, or other psychiatric diseases, (3) a history of addiction to alcohol or drugs, (4) complications of severe brain, heart, kidney, liver, and hemopoietic system diseases, (5) having any MRI contraindications, or excessive head movement during scanning. All of the healthy controls (HCs) were screened using the Clinical Diagnostic Interview Nonpatient Version without significant cognitive disorders, head trauma, or magnetic resonance imaging contraindications (24).

The People’s Hospital of Yichun City’s Medical Ethics Committee approved the reference number 2020106. All participants provided written informed consent prior to their involvement in the study.

### 2.2 Clinical evaluations

All PD patients underwent clinical assessment before the MRI scan. The study utilized the Unified Parkinson’s Disease Rating Scale (UPDRS) to assess clinical symptoms, the Modified Hoehn-Yahr staging scale to evaluate disease severity (25), and the Schwab-England scores to gauge the patient’s daily functional ability. UPDRS III was subdivided into sub-scores to assess tremor (UPDRS III items 20 and 21), rigidity (UPDRS III item 22), bradykinesia (UPDRS III items 23–26 and 31), and axial symptoms (UPDRS III items 27–30) (26). Tremor dominant (TD) scores (UPDRS II item 16 and UPDRS III item 20, 21); postural instability/gait difficulty (PIGD) scores (UPDRS II item 13–15 and UPDRS III item 29, 30). All PD patients were in the “OFF medication” state.

### 2.3 Acquisition of magnetic resonance imaging

The magnetic resonance imaging (MRI) data were obtained utilizing a 3.0 T MRI scanner (GE Discovery 750w, General Electric, Boston, MA, United States). Sagittal T1-weighted anatomical images were obtained using a three-dimensional brain volume imaging (3D BRAVO) sequence covering the entire brain. The imaging parameters were as follows: time repetition (TR)/time echo (TE) = 8.2/3.2 ms, slice thickness/gap = 1.0/0 mm, matrix = 256 × 256, field of view (FOV) = 240 × 240 mm, NEX = 1, flip angle = 12°, bandwidth = 31.25 Hz, and acquisition time = 3 min 45 s. Functional MRI data were acquired using a gradient echo echo-planar imaging sequence with the following parameters: TR/TE = 2000/25 ms, slice thickness/gap = 3.0/1.0 mm, voxel size = 3.75 × 3.75 × 3 mm^3^, field of view (FOV) = 240 × 240 mm, flip angle = 90°, matrix = 64 × 64, number of slices = 35, and 240 volumes. All participants were scanned in a supine position with their heads positioned first, and were instructed to keep their eyes closed while remaining awake and refraining from engaging in any cognitive activity. The participants were instructed to minimize movement and to use foam cushions placed symmetrically on both sides of the head to reduce motion.

### 2.4 Preprocessing of MRI data

The MRI data underwent preprocessing and analysis utilizing the Data Processing and Analysis for (Resting-State) Brain Imaging (DPABI v6.0) toolbox, which is based on the MATLAB 2018b platform (MathWorks, Inc., Natick, Massachusetts, United States). The toolbox can be accessed at http://www.rfmri.org/dpabi. The preprocessing steps include the removal of the initial 10 volumes of functional data, slice timing correction, realignment for head motion correction, tissue segmentation using voxel-based morphometry with the application of diffeomorphic anatomical registration through exponentiated Lie algebra (DARTEL) for T1-weighted anatomical data. Additionally, the functional data is co-registered to the corresponding T1 images and spatially normalized into Montreal Neurological Institute space; several nuisance variables regression is performed, including Friston 24-head motion parameters, mean frame displacement, white matter, cerebrospinal fluid, with or without global signals as covariates. The data is then resampled into 3-mm isotropic voxels, temporally bandpass filtered (0.01–0.1 Hz), and spatially smoothed with a 6-mm full width at half-maximum (FWHM) Gaussian kernel. Participants were excluded if their mean framewise displacement (FD) exceeded 0.3 mm, or if their translational/rotational movements exceeded 3 mm or 3°.

### 2.5 Analysis of functional connectivity

**Figure 1:**
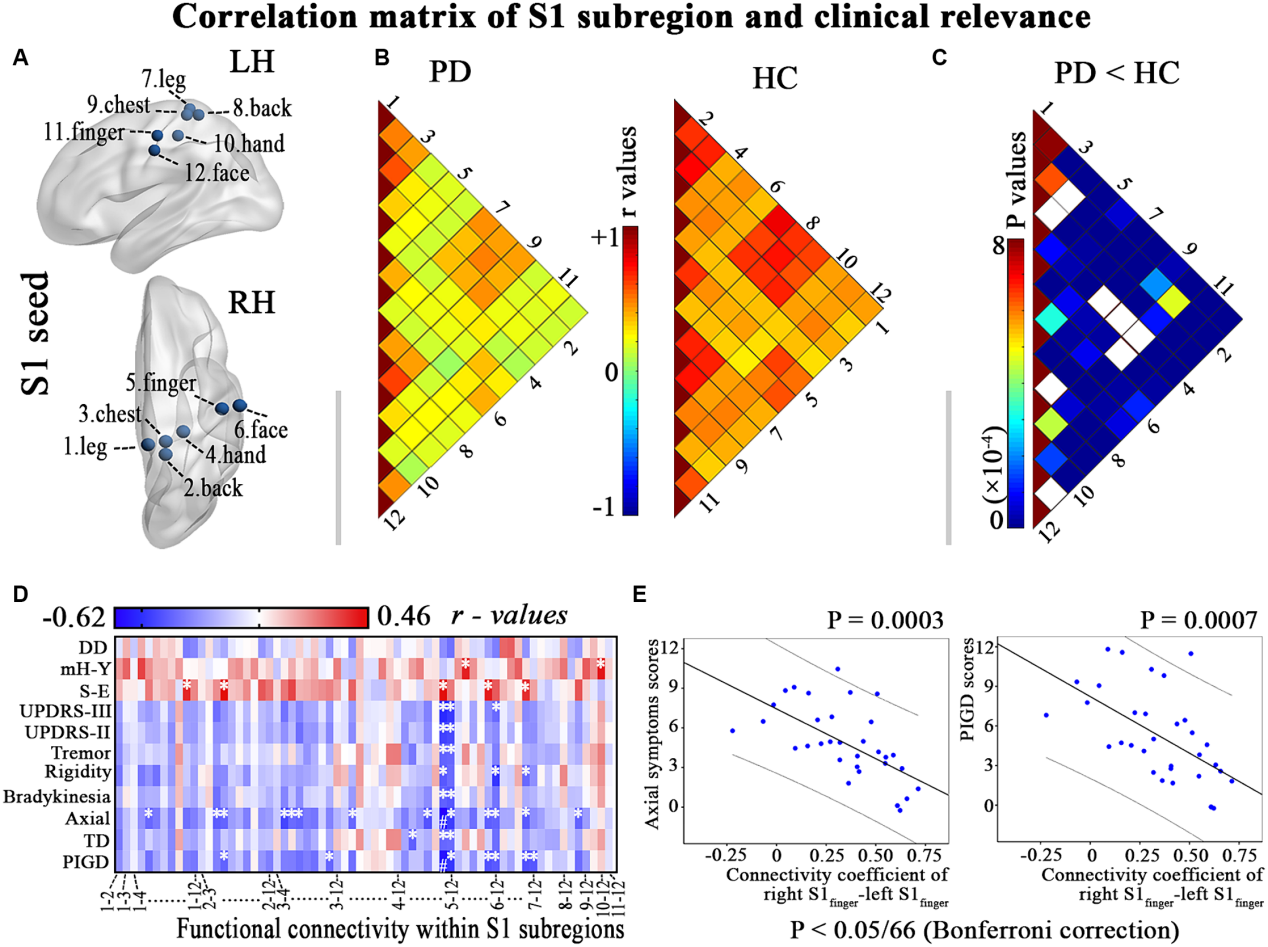
Illustrates the hypo-connectivity within S1 regions and the clinical-functional correlations that were identified in patients with PD. **(A)** S1 regions; **(B)** Correlation analysis within S1 regions of interest in PD patients and HC; **(C)** Between-group differences of interregional functional correlation by a network-based statistical approach (NBS, http://www.nitrc.org/projects/nbs/); **(D–E)** Relationship between the altered correlation matrix and the clinical feature is shown using correlation coefficients (heatmap) and scatter diagram (*p* < 0.05/66, with Bonferroni correction). DD, disease duration; LH, left hemisphere; mH-Y, Modified Hoehn and Yahr stage; PIGD, postural instability/gait difficulty; RH, right hemisphere; S-E, Schwab-England scores; TD, Tremor dominant; UPDRS, Unified Parkinson’s Disease Rating Scale. * *p* < 0.05, # *p* < 0.05/66, with Bonferroni correction.

Seed-based correlation (SBC) analysis was employed to calculate the functional connectivity. Six paired somatosensory regions of interest (ROIs) (Figure 1A) were chosen based on block-design fMRI studies (27–30) and a review article (31). The MNI coordinates of the seeds were as follows: leg (representation, $S1_{\mathrm{leg}}$, ±8,−38,68 in MNI coordinates), back ($S1_{\mathrm{back}}$: ±18, −44, 64), chest ($S1_{\mathrm{chest}}$: ±18, −36, 64), hand ($S1_{\mathrm{hand}}$: ±28, −30, 50), finger ($S1_{\mathrm{finger}}$: ±50, −16, 50), and face ($S1_{\mathrm{face}}$: ±60, −14, 40). Initially, the mean time series were extracted separately and then averaged from each 4-mm radius sphere seed centered on the coordinates mentioned above (30). Subsequently, the Pearson’s correlation of each seed with the other seeds (as a correlation matrix) or with the remaining brain voxels (as connectivity maps) was calculated. Whole brain connectivity maps derived from seed-based analysis were subjected to Fisher’s r-to-z transformation to ensure a normal distribution. Subsequently, general linear model statistics were applied to compare the connectivity matrices between individuals with PD and HCs. The connectivity maps were notably utilized to derive one-sample t-test FC masks with regression global signals, while those without regression global signals were employed for inter-group comparison.

### 2.6 Multivariate relationships between the connectivity of subregions in S1 and clinical characteristics

Partial least-squares (PLS) correlation analysis was employed to investigate the association between the modified subregional connectivity and clinical characteristics utilizing the myPLS package.^1^ Separate PLS models were constructed for each subregion in order to facilitate the identification of both shared and unique contributions of the subregions to sensorimotor symptoms. In addition, PD patient is an older population, brain features and severity of symptoms could be very much affected by age and disease duration. As such, age, sex, disease duration and education level were regressed from the imaging and data of clinical symptoms using multiple linear regression to eliminate the confounding effects of these variables. The primary steps of the analysis involve: (1) Determine the imaging matrix X (X=N×M; N is the number of included patients and M is brain voxels) and behavioral matrix Y (Y=N×B; B is the behavior score); (2) Constructing the correlation matrix R ($R=X^{T}\;Y$); (3) the R matrices ($R=X^{T}\;Y=SUV^{T}$); (4) the latent variables (LVs) that capture the maximum amount of shared information between the imaging matrix (X) and the behavioral matrix (Y). The latent variables (LVs) are represented by the singular values that form the matrix S (S=L×L, L is LV). The quantity of LVs was equal to the number of columns of behavioral matrix Y. The first LV consists of the left singular (1st column) vector of U (U=B×L), right singular (1st column) vector of V (V=M×L) and 1st column vector of matrix S. The U denotes the behavioral weights that best characterize R, and V representing voxel weights that most effectively describe R. (5) Subsequently, brain scores ($L_{X}=XV=N\times M\times M\times L$) and behavior scores ($L_{Y}=YU=N\times B\times B\times L$) were computed to delineate the individual contributions within the LV pattern.

The permutation test (randomly rearranged 1,000 times) was employed to assess the statistical significance of each LV. LVs with *p* values less than 0.05 were deemed to be statistically significant.

### 2.7 Statistical analysis

#### 2.7.1 Demographic and clinical data statistics

All demographic and clinical data were compared between groups using SPSS v22.0 (IBM, Armonk, NY, United States). The Shapiro–Wilk test was used to assess the normal distribution. The Mann–Whitney U-test or two-sample t-test was utilized to assess differences between the two groups. The chi-squared test was used to compare categorical data (gender).

#### 2.7.2 Functional connectivity statistics

The statistics module of DPABI (based on SPM12) was used to calculate within- and between-group differences in the functional connectivity data. For the connectivity maps of each S1 subregion, we initially conducted one-sample t-tests on the PD and HC groups to establish the connectivity profile of each subregion. The significance level for the within-group test was set at a false discovery rate (FDR) corrected *p* < 0.001 at the voxel level. A union FC mask was generated by combining the within-group results from both groups for each subregion. Between-group differences in connectivity maps for each S1 subregion were then measured using the general linear model within the union mask, with age, sex, disease duration and education level as covariates. The significance level was FDR-corrected with *p* < 0.05 at the voxel level and a cluster size ≥10 voxels.

Between-group differences in the correlation matrix were evaluated using a network-based statistical approach (NBS, http://www.nitrc.org/projects/nbs/).

#### 2.7.3 Correlation analysis

Partial correlation analysis was conducted to investigate the association between the region exhibiting altered connectivity (in correlation matrix or connectivity maps) and clinical features, such as UPDRS II, UPDRS-III, tremor, rigidity, bradykinesia, axial symptoms, TD, PIGD, disease duration, Schwab-England scores, and Modified Hoehn-Yahr rating scale, in patients with PD (*p* < 0.05, Bonferroni corrected), with age, sex, disease duration and education level as covariates. The PLS correlation analysis is presented in section 2.6.

## 3 Results

### 3.1 Demographic and clinical characteristics

Four patients and two HC were excluded due to translational/rotational movements of fMRI exceeding 3 mm or 3°, and two patients were excluded due to significant motion artifacts in 3D-T1WI. Finally, a total of 33 patients diagnosed with PD were included in the study, comprising 21 males and 12 females. Additionally, 34 HCs were also enrolled, consisting of 21 males and 13 females, as indicated in Table 1. There were no statistically significant differences in age, gender, and mean FD (*p* > 0.05) between the PD patients and the HCs.

**Table 1:** Demographic and clinical characteristics of the participants, mean ± standard deviation (SD).

Data	PD (*n* = 33)	HC (*n* = 34)	*p*-values
Age (years)	69.12 ± 8.86	66.59 ± 5.37	0.161^a^
Gender (M/F)	21/12	21/13	0.874^b^
Disease duration (months)	36.00 [19,69] (1,120)	n.a.	n.a.
Modified H-Y stage	2.50 [2, 3] (1,4)	n.a.	n.a.
Schwab-England scores	0.60 [0.50, 0.80] (0.30,0.90)	n.a.	n.a.
Unified Parkinson’s Disease Rating Scale (UPDRS)			
UPDRS III	18.00 [11.50, 26.00] (6,37)	n.a.	n.a.
UPDRS II	12.00 [6, 16.50] (1,28)	n.a.	n.a.
Tremor	2.33 ± 0.32	n.a.	n.a.
Rigidity	1.70 ± 0.16	n.a.	n.a.
Bradykinesia	7.00 [5.00,10.00] (2,16)	n.a.	n.a.
Axial symptoms	4.00 [3.00,7.00] (0.00,11.00)	n.a.	n.a.
TD scores	3.76 ± 0.47	n.a.	n.a.
PIGD scores	5.00 [3.00,7.00] (0.00,12.00)	n.a.	n.a.
Mean FD (mm)	0.037 ± 0.017	0.032 ± 0.023	0.877^a^

Values are presented as mean ± standard deviation (SD) or median [quartile] and (min-max). UPDRS, Unified Parkinson’s Disease Rating Scale; H-Y stage, Hoehn and Yahr stage; TD, Tremor Dominant; PIGD, postural instability/gait difficulty; FD, framewise displacement. ^a^two-sample *t*-test; ^b^chi-square test.

### 3.2 Altered correlation matrix within S1 subregions in PD

PD patients exhibit diminished functional connectivity among various S1 subregions (*p* < 0.001; Figures 1B,C). Furthermore, a negative correlation was observed between the modified correlation matrix of S1 and the scores for axial symptoms and PIGD, with a significance level of *p* < 0.05/66 after applying Bonferroni correction. This relationship is illustrated in Figures 1D,E and detailed in Supplementary Table S1. Therefore, PD patients who reported more prominent clinical features (such as axial symptoms and PIGD scores) also exhibited a more significant decrease in resting connectivity within the primary somatosensory cortex (S1).

### 3.3 Aberrant connectivity maps of the S1 subregions in PD

**Figure 2:**
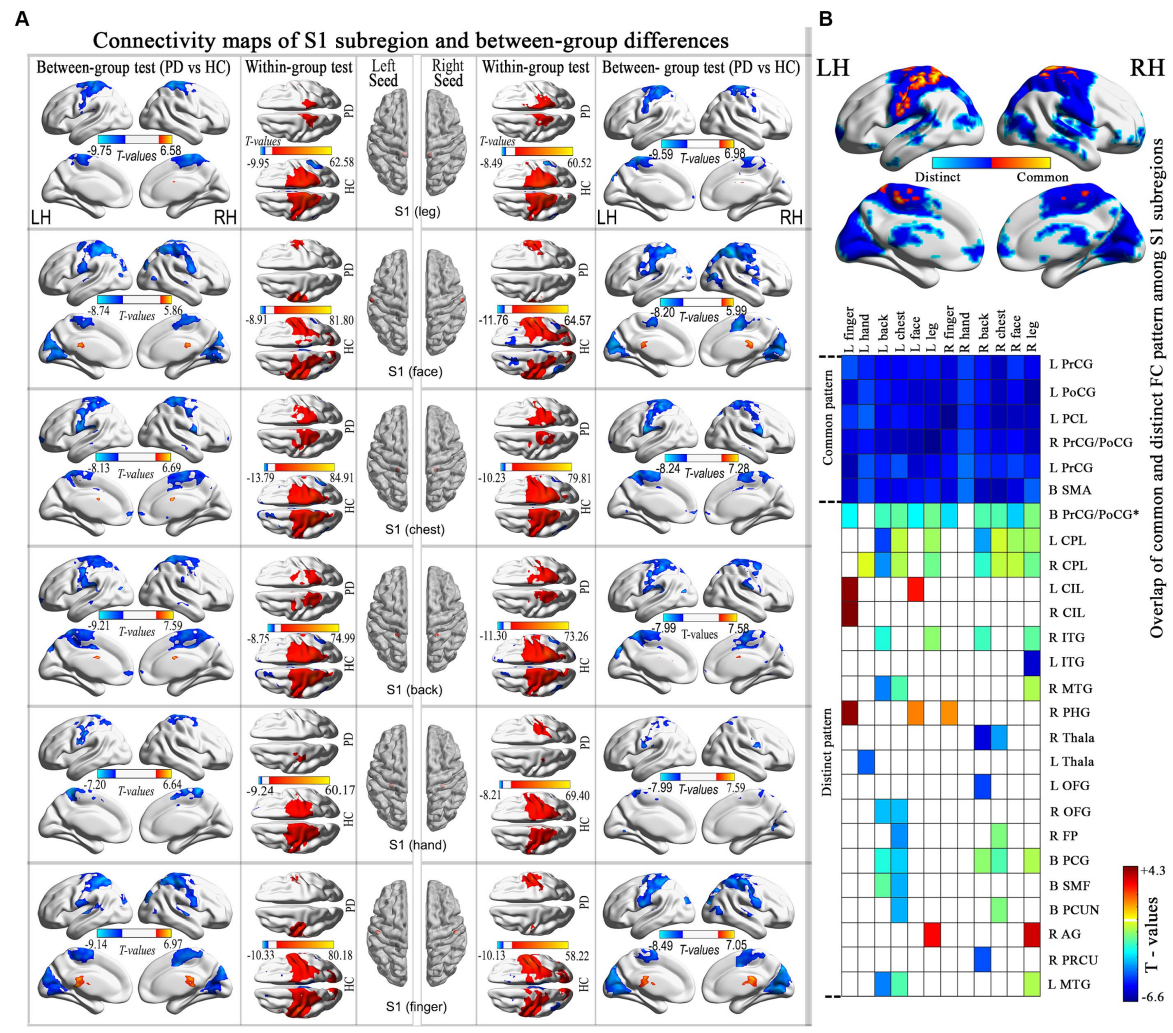
Connectivity maps of the S1 subregion and highlights the differences between groups. **(A)** Results thresholded at FDR-corrected voxel-level *p* < 0.001 (cluster size>10 voxels) in MNI152 space. **(B)** Spatial similarities and differences of altered connectivity maps among S1 subregions. PrCG, precentral gyrus; PoCG, postcentral gyrus; PCL, paracentral lobule; CPL, cerebellum posterior lobe; CIL, cerebellum inferior lobes; ITG, inferior temporal gyrus; MTG, middle temporal gyrus; PHG, parahippocampal gyrus; Thala, thalamus; OFG, orbitofrontal gyrus; FP, frontal pole; PCG, posterior cingulate gyrus; SMF, superior medial frontal; PCUN, precuneus; AG, angular gyrus; PRCU, Precuneus; MTG, middle temporal gyrus.

Statistical comparisons between groups revealed that all subregions exhibited statistically significant common hypo-connectivity in the PD group compared to the HC group (FDR corrected at the voxel level, *p* < 0.001; k ≥ 10 voxels). Distinct patterns of connectivity were also observed in PD patients (Figure 2; Supplementary Table S2).

### 3.4 The correlation between the connectivity maps of S1 subregions and the clinical signature in PD patients

**Figure 3:**
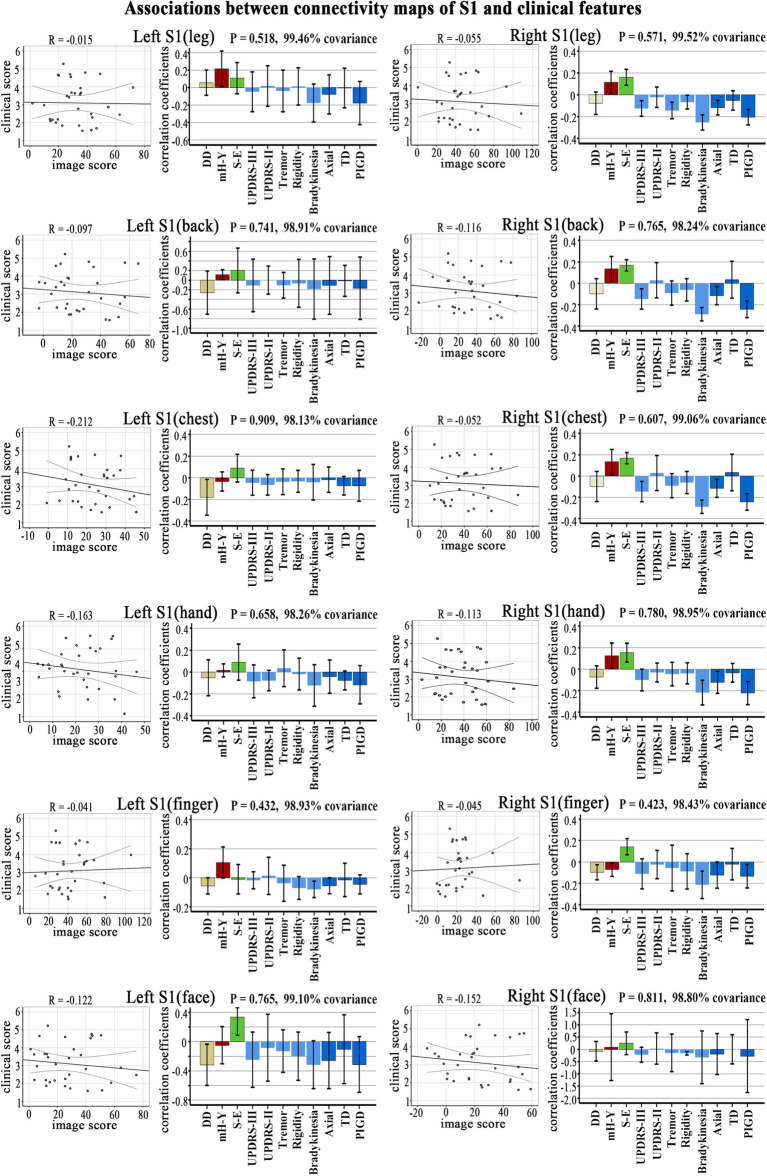
Clinical-functional signatures of connectivity maps of S1 subregions in PD. Scatter diagram: no significant correlation was found in brain scores and behavior scores of LV-1 in each subregion. Corresponding correlation coefficients shown in bar chart. Error bars indicate bootstrap estimated 95% confidence intervals of correlation strength. PIGD, postural instability/gait difficulty; UPDRS, Unified Parkinson’s Disease Rating Scale; mH-Y, Modified Hoehn and Yahr stage; TD, Tremor dominant; S-E, Schwab-England scores; DD, disease duration.

No significant correlation was observed in the PLS analysis between the FC (brain score) and clinical symptoms (behavior score) in each S1 subregion (Figure 3).

## 4 Discussion

This study examined the connectivity abnormalities in S1 subregions in PD and subsequently established a connection between these modified connectivity patterns and the clinical characteristics of PD using partial correlation or PLS analysis. We found that (1) the disrupted correlation matrix in S1 subregions showed a negative correlation with axial symptom scores and PIGD scores; (2) there was a common hypo-connectivity with S1 subregions mainly in the sensorimotor network, while distinct hypo-connectivity with the S1 subregions was mainly observed in the default mode network and cerebellum posterior lobe; (3) the disrupted connectivity maps of S1 subregions did not demonstrate any clinical correlation.

### 4.1 The hypo-connectivity matrix within the S1 subregions in PD

Our study has identified evidence indicating hypo-connectivity between S1 subregions in PD patients. Alterations in sensory function have been documented in PD patients (32). One potential mechanism is that the denervation of basal ganglia by disease-related dopaminergic dysfunction leads to a loss of response specificity, causing the transmission of noisy and poorly differentiated information to cortical regions (19). Disorders of sensor-motor integration in patients with Parkinson’s disease may also contribute to this phenomenon, involving alterations in the relationship between sensory input and motor output (33). Previous research has demonstrated that the connectivity of M1 is affected by disrupted motor pathways in PD patients, which is attributed to dopamine deficits (34). The S1 plays a significant role in providing activating input to the motor cortex and contributes to the integration of sensory and motor signals essential for proficient movement. The results of our study provide additional evidence for the idea that sensorimotor integration dysfunction in PD patients significantly influences the modulation of motor output, the process of learning, the modification of motor skills to adapt to the environment, and ultimately contributes to motor dysfunction (17).

The correlation analysis revealed a significant association between reduced S1 matrix connectivity and both axial symptoms score and PIGD scores. This finding provides additional evidence for the hypothesis that S1 internal hypo-connectivity is a significant factor in the motor symptoms experienced by patients with Parkinson’s disease. Patients with PD frequently display either symptoms predominantly characterized by postural instability and gait difficulty (PIGD) or experience a loss of balance attributed to compromised postural reflexes, phenomena that are thoroughly comprehended. Previous research has established that in PIGD, there is a disrupted connectivity between two SMN subnetworks (35). The axial symptoms of Parkinson’s disease, such as freezing of gait, postural instability, trunk posture alterations, and balance difficulties, exert a substantial influence on the quality of life of affected individuals. Impaired sensor-motor integration is also a significant factor contributing to the decline of motor skills in PD patients (15). Research on the effects of pedunculopontine nucleus area (PPNa) deep brain stimulation (DBS) treatment has demonstrated the potential for improvement in axial symptoms in PD patients, as well as the restoration of sensory-related functional connections (36, 37).

### 4.2 Distinct patterns of connectivity with other functional networks are observed in the S1 subregion of PD patients

In the examination of connectivity maps of S1 subregions, in addition to the typical hypo-connectivity pattern in the sensorimotor network, a noticeable altered pattern was observed in the connection between the S1chest and S1back subregions, primarily situated in the DMN. As early as 1970, Jones and Powell identified a fiber connection linking S1 and S2, which subsequently traverses BA5-BA7 before reaching the heteromodal regions, now recognized as the DMN. The existence of dorsal (e.g., by the dorsal anterior cingulate cortex) and ventral (e.g., by the operculum parietal and frontoinsular) functional streams of S1 has been validated through functional MRI studies (38). Simultaneously, the posterior cingulate cortex, which is a component of the DMN, also projects to the SMN and basal ganglia (BG) centers, contributing to the modulation of movement (39). The brain activity patterns in healthy individuals revealed the involvement of a specific network (DMN) during control trials and its disengagement during executive trials. The impairment of the DMN in PD has also been evidenced (12). Hence, an impairment in the co-activation of the DMN and SMN could potentially play a role in the motor impairments observed in PD.

This study emphasizes the modified functional connectivity pattern among S1 subregions in individuals with Parkinson’s disease. Nevertheless, it is important to acknowledge several limitations. Firstly, our findings are derived from initial observational studies utilizing cross-sectional data. The role of this S1 hypoconnectivity should be validated in larger patient cohort studies in the future. Secondly, as a prospective collection, retrospective study, which was not well thought out at the time. So, this study did not incorporate a dedicated sensory function assessment for patients with Parkinson’s disease. In addition to the primary indicators, individuals with Parkinson’s disease also experience a range of non-motor symptoms such as sensory impairments, sleep disturbances, autonomic dysfunction, and mental disorders, all of which can significantly impact their quality of life. In future research, somatosensory evoked potentials (SEPs) could be elicited in PD patients to assess evoked potentials. Despite the discussion of resting state functional networks, such as the DMN, this study did not identify a correlation between hypo-connectivity of the S1 subregions and clinical assessment scales. Subsequent research endeavors may focus on examining the clinical significance of the correlation between the S1 subregion and individual RSNs.

## 5 Conclusion

In conclusion, our study found a notable decrease in functional connectivity within the S1 subregions of PD patients. Furthermore, the initial findings of this study indicate that internal hypo-connectivity in the S1 region may significantly contribute to the motor symptoms observed in PD patients. Our study offers new insights into the neurobiological mechanisms underlying S1 dysfunction in PD.

## Data availability statement

The raw data supporting the conclusions of this article will be made available by the authors, without undue reservation.

## Ethics statement

The studies involving humans were approved by The People’s Hospital of Yichun City’s Medical Ethics Committee. The studies were conducted in accordance with the local legislation and institutional requirements. The participants provided their written informed consent to participate in this study.

## Author contributions

YW: Conceptualization, Funding acquisition, Investigation, Project administration, Resources, Software, Supervision, Visualization, Writing – original draft, Writing – review & editing. TG: Data curation, Investigation, Writing – original draft, Writing – review & editing. NT: Data curation, Investigation, Writing – original draft, Writing – review & editing. YZ: Data curation, Investigation, Writing – original draft, Writing – review & editing. HM: Investigation, Writing – original draft, Writing – review & editing. WY: Data curation, Writing – original draft, Writing – review & editing. WL: Data curation, Writing – original draft, Writing – review & editing. FZ: Conceptualization, Formal analysis, Funding acquisition, Investigation, Project administration, Resources, Software, Supervision, Visualization, Writing – original draft, Writing – review & editing.

## Footnotes

^1^ https://github.com/danizoeller/myPLS
