# Supplementary material for: Hypo-connectivity of the primary somatosensory cortex in Parkinson’s disease: a resting-state functional MRI study
Source: Front Neurol. 2024 Apr 30;15:1361063. doi: 10.3389/fneur.2024.1361063 (PMC11091379; doi:10.3389/fneur.2024.1361063)
Supplement: Supplementary file 1 [file Table_1.docx]

**Table S1.** Relationship between the connectivity coefficient within S1 regions and clinical features.

| P values | DD | mH-Y | S-E | UPDRS-III | UPDRS-II | Tremor | Rigidity | Bradykinesia | axial symptoms | TD score | PIGD |
| --- | --- | --- | --- | --- | --- | --- | --- | --- | --- | --- | --- |
| S1S2 | 0.701 | 0.331 | 0.479 | 0.211 | 0.157 | 0.131 | 0.070 | 0.281 | 0.114 | 0.077 | 0.137 |
| S1S3 | 0.467 | 0.126 | 0.851 | 0.846 | 0.665 | 0.481 | 0.629 | 0.844 | 0.422 | 0.435 | 0.418 |
| S1S4 | 0.474 | 0.862 | 0.847 | 0.794 | 0.748 | 0.713 | 0.863 | 0.879 | 0.719 | 0.786 | 0.844 |
| S1S5 | 0.421 | 0.060 | 0.878 | 0.272 | 0.56 | 0.494 | 0.08 | 0.249 | 0.153 | 0.227 | 0.397 |
| S1S6 | 0.766 | 0.290 | 0.198 | 0.188 | 0.312 | 0.669 | 0.157 | 0.323 | 0.043* | 0.564 | 0.059 |
| S1S7 | 0.528 | 0.514 | 0.621 | 0.287 | 0.286 | 0.517 | 0.060 | 0.307 | 0.079 | 0.325 | 0.225 |
| S1S8 | 0.729 | 0.493 | 0.487 | 0.759 | 0.556 | 0.594 | 0.501 | 0.826 | 0.396 | 0.420 | 0.498 |
| S1S9 | 0.636 | 0.677 | 0.399 | 0.224 | 0.137 | 0.210 | 0.171 | 0.293 | 0.100 | 0.119 | 0.115 |
| S1S10 | 0.862 | 0.336 | 0.673 | 0.449 | 0.643 | 0.318 | 0.507 | 0.530 | 0.824 | 0.393 | 0.782 |
| S1S11 | 0.084 | 0.964 | 0.030* | 0.200 | 0.212 | 0.516 | 0.497 | 0.362 | 0.056 | 0.291 | 0.087 |
| S1S12 | 0.108 | 0.941 | 0.241 | 0.192 | 0.190 | 0.290 | 0.210 | 0.251 | 0.095 | 0.234 | 0.071 |
| S2S3 | 0.373 | 0.852 | 0.856 | 0.379 | 0.478 | 0.513 | 0.890 | 0.658 | 0.239 | 0.509 | 0.612 |
| S2S4 | 0.766 | 0.757 | 0.638 | 0.968 | 0.978 | 0.908 | 0.762 | 0.661 | 0.893 | 0.975 | 0.825 |
| S2S5 | 0.417 | 0.979 | 0.273 | 0.057 | 0.141 | 0.186 | 0.112 | 0.057 | 0.029* | 0.121 | 0.093 |
| S2S6 | 0.261 | 0.978 | 0.009* | 0.156 | 0.174 | 0.672 | 0.122 | 0.202 | 0.037* | 0.478 | 0.035* |
| S2S7 | 0.73 | 0.348 | 0.570 | 0.324 | 0.317 | 0.457 | 0.538 | 0.360 | 0.193 | 0.319 | 0.309 |
| S2S8 | 0.928 | 0.280 | 0.514 | 0.854 | 0.803 | 0.951 | 0.451 | 0.835 | 0.279 | 0.931 | 0.468 |
| S2S9 | 0.988 | 0.636 | 0.257 | 0.371 | 0.301 | 0.550 | 0.141 | 0.662 | 0.090 | 0.417 | 0.112 |
| S2S10 | 0.930 | 0.270 | 0.929 | 0.953 | 0.834 | 0.935 | 0.812 | 0.712 | 0.904 | 0.768 | 0.902 |
| S2S11 | 0.169 | 0.827 | 0.098 | 0.381 | 0.386 | 0.557 | 0.411 | 0.488 | 0.155 | 0.403 | 0.168 |
| S2S12 | 0.324 | 0.621 | 0.169 | 0.294 | 0.250 | 0.613 | 0.476 | 0.329 | 0.193 | 0.436 | 0.090 |
| S3S4 | 0.954 | 0.448 | 0.714 | 0.571 | 0.398 | 0.861 | 0.595 | 0.540 | 0.476 | 0.679 | 0.54 |
| S3S5 | 0.551 | 0.883 | 0.183 | 0.062 | 0.152 | 0.442 | 0.233 | 0.106 | 0.011* | 0.282 | 0.088 |
| S3S6 | 0.615 | 0.435 | 0.066 | 0.305 | 0.389 | 0.741 | 0.283 | 0.438 | 0.048* | 0.781 | 0.085 |
| S3S7 | 0.651 | 0.809 | 0.457 | 0.109 | 0.055 | 0.404 | 0.208 | 0.134 | 0.035* | 0.298 | 0.053 |
| S3S8 | 0.742 | 0.569 | 0.421 | 0.257 | 0.182 | 0.157 | 0.248 | 0.549 | 0.083 | 0.155 | 0.088 |
| S3S9 | 0.605 | 0.408 | 0.334 | 0.262 | 0.167 | 0.311 | 0.321 | 0.616 | 0.068 | 0.298 | 0.056 |
| S3S10 | 0.618 | 0.823 | 0.275 | 0.701 | 0.499 | 0.919 | 0.615 | 0.691 | 0.531 | 0.98 | 0.509 |
| S3S11 | 0.188 | 0.932 | 0.224 | 0.170 | 0.117 | 0.303 | 0.581 | 0.289 | 0.097 | 0.250 | 0.041* |
| S3S12 | 0.832 | 0.211 | 0.378 | 0.879 | 0.771 | 0.538 | 0.425 | 0.780 | 0.326 | 0.576 | 0.306 |
| S4S5 | 0.980 | 0.458 | 0.842 | 0.683 | 0.681 | 0.997 | 0.530 | 0.626 | 0.478 | 0.791 | 0.916 |
| S4S6 | 0.140 | 0.387 | 0.121 | 0.171 | 0.125 | 0.957 | 0.334 | 0.178 | 0.044* | 0.953 | 0.057 |
| S4S7 | 0.524 | 0.481 | 0.646 | 0.677 | 0.787 | 0.335 | 0.337 | 0.435 | 0.796 | 0.298 | 0.979 |
| S4S8 | 0.959 | 0.686 | 0.631 | 0.738 | 0.768 | 0.590 | 0.944 | 0.825 | 0.657 | 0.688 | 0.838 |
| S4S9 | 0.939 | 0.671 | 0.444 | 0.766 | 0.734 | 0.785 | 0.897 | 0.680 | 0.660 | 0.751 | 0.688 |
| S4S10 | 0.953 | 0.209 | 0.433 | 0.797 | 0.977 | 0.961 | 0.514 | 0.890 | 0.591 | 0.978 | 0.770 |
| S4S11 | 0.769 | 0.622 | 0.573 | 0.770 | 0.831 | 0.206 | 0.504 | 0.758 | 0.771 | 0.259 | 0.933 |
| S4S12 | 0.478 | 0.948 | 0.436 | 0.771 | 0.721 | 0.202 | 0.523 | 0.939 | 0.304 | 0.261 | 0.511 |
| S5S6 | 0.931 | 0.786 | 0.932 | 0.206 | 0.146 | 0.220 | 0.673 | 0.341 | 0.090 | 0.257 | 0.129 |
| S5S7 | 0.936 | 0.350 | 0.903 | 0.205 | 0.484 | 0.078 | 0.490 | 0.361 | 0.297 | 0.032* | 0.438 |
| S5S8 | 0.808 | 0.220 | 0.619 | 0.291 | 0.381 | 0.617 | 0.389 | 0.267 | 0.154 | 0.407 | 0.450 |
| S5S9 | 0.578 | 0.825 | 0.530 | 0.098 | 0.124 | 0.446 | 0.356 | 0.134 | 0.021* | 0.304 | 0.133 |
| S5S10 | 0.833 | 0.786 | 0.590 | 0.947 | 0.993 | 0.634 | 0.423 | 0.847 | 0.589 | 0.606 | 0.794 |
| S5S11 | 0.136 | 0.475 | 0.012* | 0.003* | 0.009* | 0.035* | 0.024* | 0.034* | 0.0003# | 0.042* | 0.0007# |
| S5S12 | 0.059 | 0.757 | 0.19 | 0.007* | 0.013* | 0.041* | 0.049* | 0.024* | 0.008* | 0.048* | 0.006* |
| S6S7 | 0.600 | 0.296 | 0.852 | 0.413 | 0.787 | 0.739 | 0.987 | 0.775 | 0.223 | 0.731 | 0.366 |
| S6S8 | 0.789 | 0.024* | 0.936 | 0.879 | 0.885 | 0.275 | 0.706 | 0.945 | 0.268 | 0.415 | 0.650 |
| S6S9 | 0.601 | 0.292 | 0.706 | 0.239 | 0.232 | 0.854 | 0.874 | 0.378 | 0.057 | 0.926 | 0.156 |
| S6S10 | 0.641 | 0.782 | 0.852 | 0.922 | 0.990 | 0.419 | 0.554 | 0.707 | 0.583 | 0.396 | 0.731 |
| S6S11 | 0.234 | 0.823 | 0.022* | 0.117 | 0.197 | 0.476 | 0.462 | 0.286 | 0.032* | 0.555 | 0.019 |
| S6S12 | 0.088 | 0.769 | 0.105 | 0.046 | 0.126 | 0.093 | 0.006* | 0.065 | 0.036* | 0.053 | 0.026* |
| S7S8 | 0.133 | 0.924 | 0.485 | 0.815 | 0.802 | 0.691 | 0.622 | 0.826 | 0.261 | 0.79 | 0.464 |
| S7S9 | 0.113 | 0.265 | 0.694 | 0.487 | 0.321 | 0.966 | 0.532 | 0.544 | 0.261 | 0.749 | 0.240 |
| S7S10 | 0.566 | 0.118 | 0.936 | 0.625 | 0.980 | 0.746 | 0.431 | 0.650 | 0.813 | 0.849 | 0.833 |
| S7S11 | 0.155 | 0.766 | 0.036* | 0.114 | 0.148 | 0.291 | 0.045* | 0.263 | 0.028* | 0.179 | 0.019* |
| S7S12 | 0.786 | 0.617 | 0.364 | 0.154 | 0.140 | 0.239 | 0.140 | 0.330 | 0.135 | 0.390 | 0.023* |
| S8S9 | 0.958 | 0.741 | 0.667 | 0.379 | 0.365 | 0.338 | 0.936 | 0.574 | 0.217 | 0.327 | 0.446 |
| S8S10 | 0.822 | 0.565 | 0.438 | 0.470 | 0.522 | 0.263 | 0.472 | 0.459 | 0.415 | 0.256 | 0.856 |
| S8S11 | 0.750 | 0.437 | 0.798 | 0.562 | 0.533 | 0.297 | 0.857 | 0.630 | 0.458 | 0.243 | 0.462 |
| S8S12 | 0.456 | 0.177 | 0.900 | 0.875 | 0.929 | 0.765 | 0.245 | 0.592 | 0.725 | 0.721 | 0.488 |
| S9S10 | 0.247 | 0.376 | 0.822 | 0.507 | 0.391 | 0.439 | 0.682 | 0.401 | 0.489 | 0.500 | 0.836 |
| S9S11 | 0.185 | 0.740 | 0.091 | 0.194 | 0.188 | 0.313 | 0.629 | 0.427 | 0.037* | 0.232 | 0.051 |
| S9S12 | 0.824 | 0.109 | 0.656 | 0.667 | 0.502 | 0.637 | 0.937 | 0.932 | 0.204 | 0.727 | 0.192 |
| S10S11 | 0.514 | 0.286 | 0.730 | 0.825 | 0.777 | 0.416 | 0.566 | 0.791 | 0.740 | 0.652 | 0.892 |
| S10S12 | 0.907 | 0.019* | 0.381 | 0.382 | 0.459 | 0.216 | 0.299 | 0.379 | 0.824 | 0.360 | 0.654 |
| S11S12 | 0.674 | 0.673 | 0.719 | 0.661 | 0.958 | 0.572 | 0.893 | 0.933 | 0.542 | 0.660 | 0.702 |

Note: DD, disease duration; mH-Y, Modified Hoehn and Yahr stage; PIGD, postural instability/gait difficulty; S-E, Schwab-england scores; TD, Tremor dominant; UPDRS, Unified Parkinson’s Disease Rating Scale. S1: right ${S1}_{leg}$; S2: right ${S1}_{back}$; S3: right ${S1}_{chest}$; S4: right ${S1}_{hand}$; S5: right ${S1}_{finger}$; S6: right ${S1}_{face}$; S7: left ${S1}_{leg}$; S8: left ${S1}_{back}$; S9: left ${S1}_{chest}$; S10: left ${S1}_{hand}$; S11: left ${S1}_{finger}$; S12: left ${S1}_{face}$. * *P* < 0.05; # *P* < 0.05/66, with Bonferroni correction.

**Table S2.** Differences between PD and HC participants for connectivity maps of S1 subregions.

| PD *vs.* HC | Hemisphere | MNI (mm) | | | Peak t-values | Cluster size | Size effect* |
| --- | --- | --- | --- | --- | --- | --- | --- |
| Brain region |  | X | Y | Z |  |  |  |
| **Seed: Right** $\mathbf{S1}_{\mathbf{leg}}$ **(8, -38, 68）** | | | | | | | |
| Cerebellum inferior | LH | -18 | -54 | -57 | -5.775 | 46 | 1.465 |
| Cerebellum inferior | RH | 15 | -63 | -60 | -6.645 | 40 | 1.478 |
| Inferior temporal gyrus | LH | -45 | -42 | -27 | -6.383 | 21 | 1.877 |
| Medial frontal gyrus | LH | -3 | 60 | 0 | -6.854 | 61 | 1.459 |
| Cuneus | LH | -12 | -87 | 39 | -8.851 | 95 | 2.077 |
| Postcentral/precentral gyrus | RH/LH | 18 | -27 | 75 | -9.595 | 3501 | 1.959 |
| Precentral gyrus | RH | 57 | -6 | 24 | -6.458 | 99 | 1.444 |
| Cingulate gyrus | RH | 18 | -33 | 27 | 6.978 | 33 | -1.874 |
| **Seed: Right** $\mathbf{S1}_{\mathbf{face}}$ **(60, -14, 40）** | | | | | | | |
| Cerebellum posterior lobe | LH | -12 | -78 | -48 | -6.718 | 52 | 1.717 |
| Postcentral/precentral gyrus/cuneus/lingual gyrus | RH/LH | 36 | -48 | 54 | -8.202 | 5953 | 2.232 |
| Postcentral/precentral gyrus | LH | -42 | -36 | 42 | -7.859 | 2320 | 1.969 |
| Superior temporal gyrus | RH | 57 | -21 | -3 | -4.926 | 25 | 1.168 |
| Corpus callosum | RH/LH | -12 | -36 | 9 | 5.995 | 187 | -1.656 |
| **Seed: Right** $\mathbf{S1}_{\mathbf{chest}}$**(18, -36, 64)** | | | | | | | |
| Cerebellum inferior | LH | -18 | -54 | -54 | -6.318 | 27 | 1.456 |
| Middle temporal gyrus | RH | 60 | -18 | -12 | -4.639 | 46 | 1.263 |
| Medial frontal gyrus | LH | -3 | 60 | -3 | -7.589 | 153 | 1.685 |
| Lingual gyrus | LH | -6 | -63 | 0 | -7.788 | 37 | 1.711 |
| Postcentral/precentral gyrus | RH/LH | 9 | -9 | 75 | -8.425 | 4823 | 1.978 |
| Anterior cingulate gyrus | RH/LH | 24 | -42 | 24 | 7.286 | 102 | -1.790 |
| **Seed: Right** $\mathbf{S1}_{\mathbf{back}}$**(18, -44, 64)** | | | | | | | |
| Cerebellum posterior lobe | LH | -21 | -72 | -54 | -7.990 | 182 | 1.850 |
| Cerebellum posterior lobe | RH | 15 | -63 | -60 | -7.177 | 50 | 1.701 |
| Inferior frontal gyrus | LH | -24 | 15 | -15 | -5.943 | 29 | 1.588 |
| Putamen | RH | 30 | -12 | 6 | -5.846 | 45 | 1.405 |
| Postcentral/precentral gyrus | RH/LH | 63 | 9 | 6 | -7.675 | 5060 | 1.378 |
| Medial frontal gyrus | RH/LH | 3 | 57 | -3 | -5.521 | 108 | 1.817 |
| Thalamus | RH | 15 | -24 | -3 | -7.258 | 21 | 1.347 |
| Insula | LH | -33 | -18 | 6 | -6.041 | 28 | 1.748 |
| **Seed: Right** $\mathbf{S1}_{\mathbf{hand}}$**(28, -30, 50)** | | | | | | | |
| Calcarine | RH | 15 | -75 | 6 | -6.360 | 161 | 1.509 |
| Postcentral/precentral gyrus | RH/LH | 60 | 9 | 15 | -6.609 | 1829 | 1.652 |
| **Seed: Right** $\mathbf{S1}_{\mathbf{finger}}$**(50, -16, 50)** | | | | | | | |
| Precuneus/calcarine/lingual | RH/LH | -18 | -72 | 42 | -8.489 | 2612 | 1.903 |
| Insula | RH | 30 | -9 | 3 | -5.452 | 64 | 1.348 |
| Corpus Callosum | RH/LH | 6 | -27 | 18 | 7.048 | 253 | -1.711 |
| Postcentral/precentral gyrus | RH/LH | -12 | -12 | 75 | -8.296 | 5012 | 1.945 |
| **Seed: Left** $\mathbf{S1}_{\mathbf{leg}}$ **(-8, -38, 68)** | | | | | | | |
| Cerebellum inferior | LH | -18 | -66 | -57 | -6.504 | 42 | 1.656 |
| Cerebellum inferior | RH | 12 | -63 | -60 | -7.721 | 39 | 1.631 |
| Cuneus | LH | -3 | -84 | 30 | -6.062 | 34 | 1.489 |
| Postcentral/precentral gyrus | RH/LH | 18 | -27 | 75 | -9.750 | 3889 | 2.136 |
| **Seed: Left** $\mathbf{S1}_{\mathbf{face}}$ **(-60, -14, 40)** | | | | | | | |
| Cerebellum posterior lobe | LH | -12 | -90 | -33 | 4.819 | 25 | -1.138 |
| Postcentral/precentral gyrus/cuneus/lingual gyrus | RH/LH | 36 | -51 | 66 | -8.741 | 6633 | 2.091 |
| Middle occipital gyrus | RH | 51 | -69 | 9 | -6.705 | 41 | 1.404 |
| Superior temporal gyrus | LH | -48 | -42 | 18 | -6.658 | 74 | 1.592 |
| **Seed: Left** $\mathbf{S1}_{\mathbf{chest}}$ **(-18, -36, 64）** | | | | | | | |
| Cerebellum inferior | RH | 15 | -60 | -60 | -6.283 | 20 | 1.315 |
| Cerebellum inferior | LH | -21 | -45 | -54 | -6.563 | 31 | 1.454 |
| Middle temporal gyrus | RH | 60 | -21 | -9 | -5.789 | 43 | 1.495 |
| Middle frontal gyrus | RH | 33 | 60 | 0 | -6.191 | 55 | 1.699 |
| Middle temporal gyrus | LH | -60 | -21 | -9 | -5.099 | 23 | 1.907 |
| Medial frontal gyrus | RH/LH | -27 | 60 | -6 | -6.885 | 290 | 1.462 |
| Postcentral/precentral gyrus/ medial frontal gyrus | RH/LH | -42 | -18 | 36 | -7.931 | 4473 | 1.339 |
| Precentral gyrus | RH | 63 | 9 | 6 | -8.135 | 149 | 1.674 |
| Superior frontal gyrus | R/L | 3 | 63 | 27 | -5.045 | 58 | 1.373 |
| Precuneus | LH | -3 | -69 | 39 | -5.203 | 46 | 1.160 |
| **Seed: Left** $\mathbf{S1}_{\mathbf{back}}$ **(-18, -44, 64)** | | | | | | | |
| Cerebellum posterior lobe | LH | -18 | -48 | -51 | -7.709 | 251 | 1.803 |
| Cerebellum posterior lobe | RH | 15 | -60 | -60 | -6.413 | 113 | 1.608 |
| Inferior temporal gyrus |  |  |  |  |  |  |  |
| Cerebellum anterior lobe | RH | 21 | -36 | -30 | -6.087 | 27 | 1.517 |
| Cerebellum anterior lobe | LH | -27 | -33 | -21 | -6.695 | 29 | 1.793 |
| Middle temporal gyrus | RH | 60 | -18 | -6 | -6.037 | 114 | 1.438 |
| Middle temporal gyrus | LH | -57 | -21 | -6 | -5.416 | 52 | 1.397 |
| Medial frontal gyrus | LH | -24 | 63 | -6 | -8.520 | 214 | 1.664 |
| Left Cerebrum |  |  |  |  |  |  |  |
| Postcentral/precentral gyrus/cuneus/lingual gyrus/medial frontal gyrus | RH/LH | 18 | -45 | 72 | -8.422 | 5372 | 1.579 |
| Postcentral gyrus | RH | 66 | -27 | 27 | -5.197 | 79 | 1.307 |
| **Seed: Left** $\mathbf{S1}_{\mathbf{hand}}$ **(-28, -30, 50)** | | | | | | | |
| Postcentral/precentral gyrus/cuneus/lingual gyrus/medial frontal gyrus | RH/LH | -6 | -42 | 66 | -7.198 | 2747 | 1.634 |
| **Seed: Left** $\mathbf{S1}_{\mathbf{finger}}$ **(-50, -16, 50)** | | | | | | | |
| Cerebellum posterior lobe | RH | 12 | -93 | -33 | 6.031 | 110 | -1.438 |
| Cerebellum posterior lobe | LH | -9 | -90 | -36 | 4.958 | 38 | -1.219 |
| Midbrain |  | 12 | -15 | -15 | 5.792 | 49 | -1.650 |
| Precuneus/cuneus/calcarine/lingual gyrus | RH/LH | 18 | -87 | 21 | -8.003 | 1860 | 1.648 |
| Middle occipital gyrus | RH | 42 | -75 | 3 | -6.174 | 50 | 1.461 |
| Postcentral/precentral gyrus/ medial frontal gyrus | RH/LH | -24 | -48 | 57 | -9.140 | 5105 | 1.988 |
| Corpus Callosum | RH/LH | -9 | -36 | 9 | 6.974 | 279 | -2.034 |

Note: *Cohen's d values
